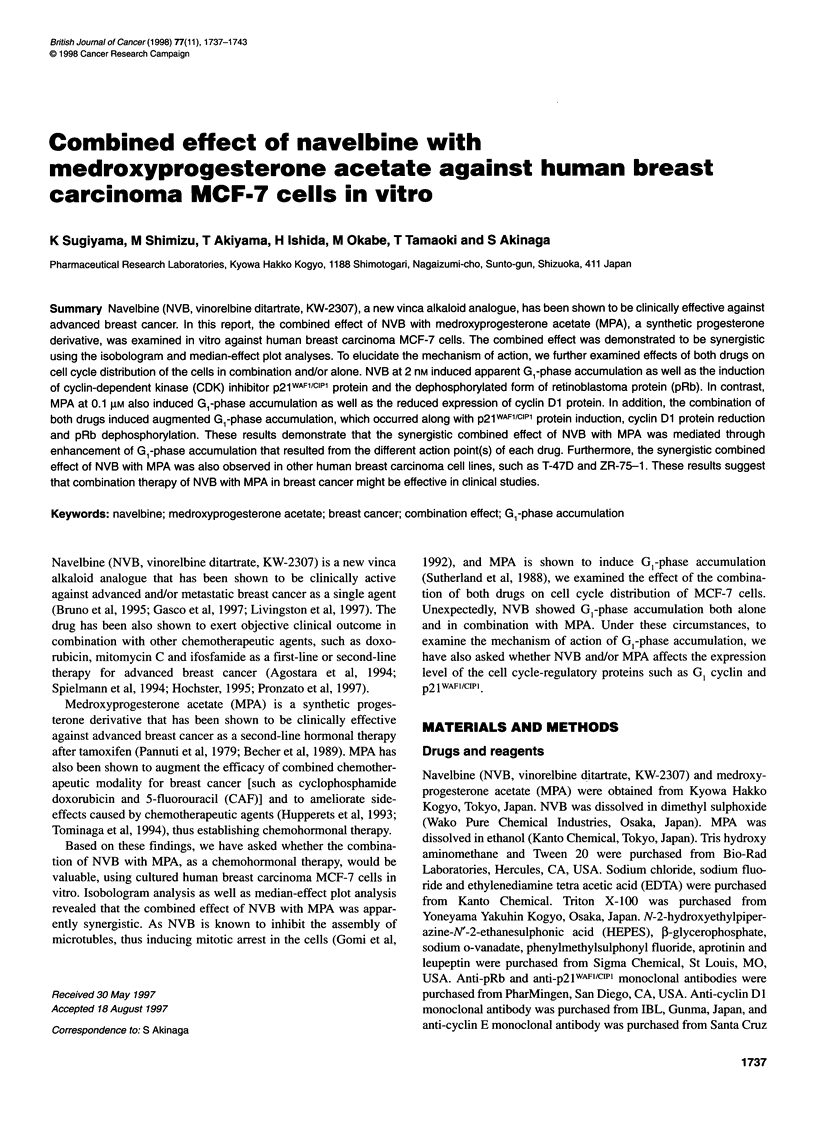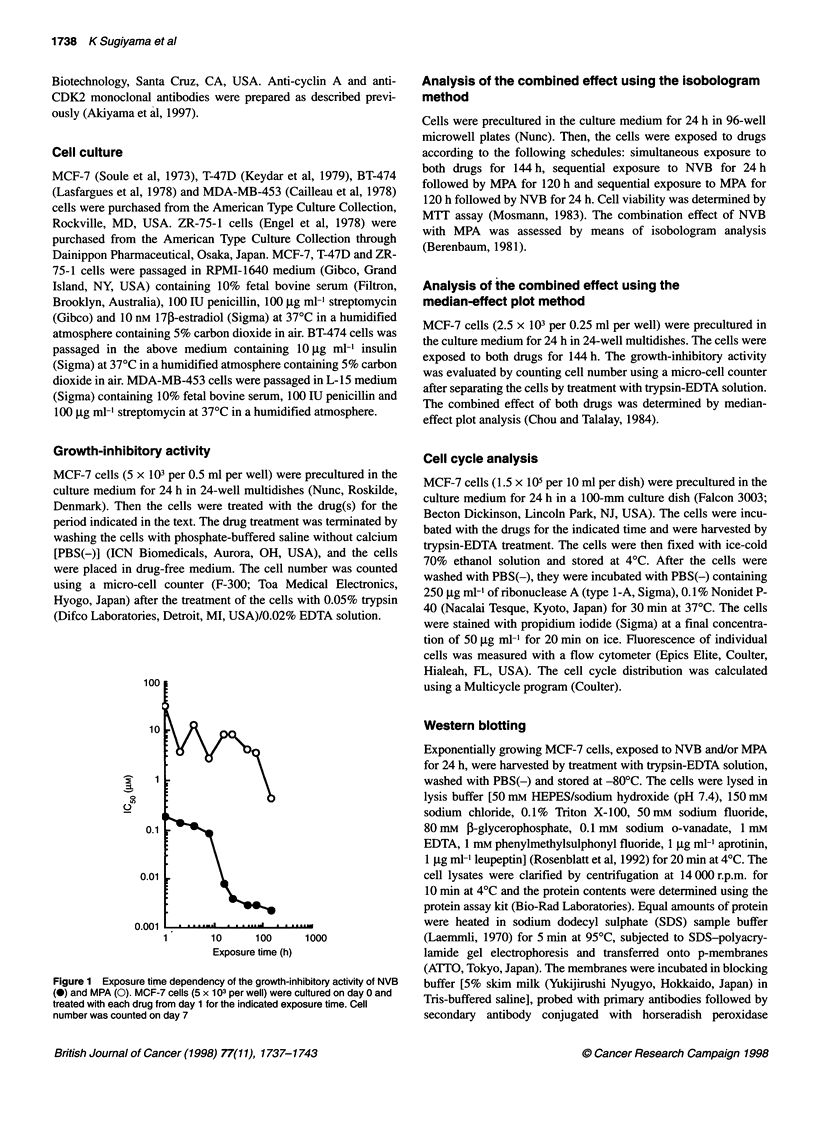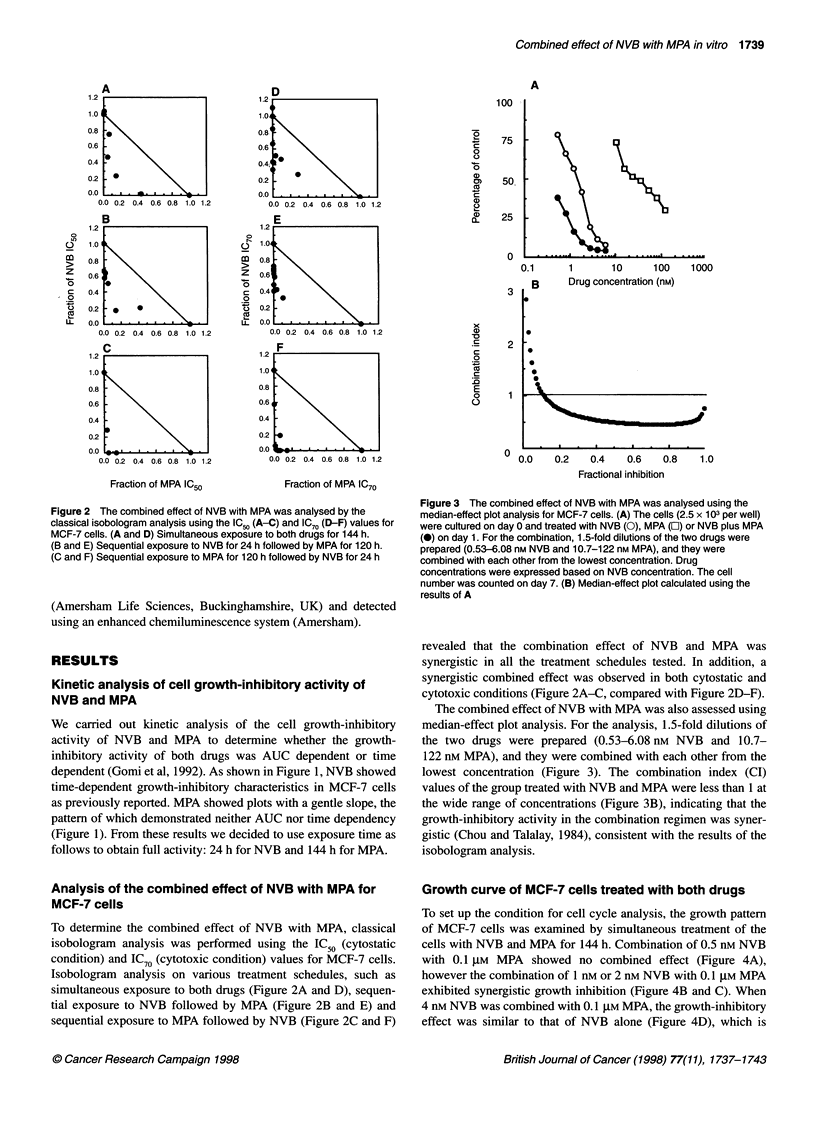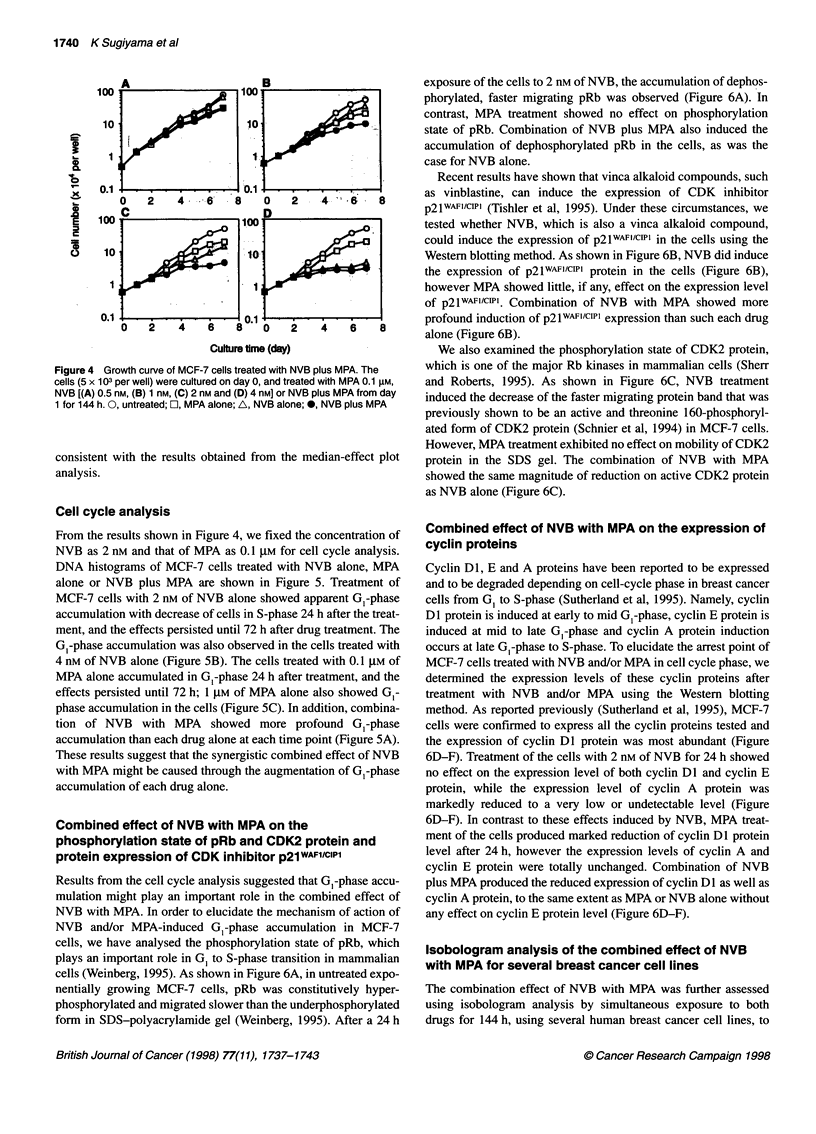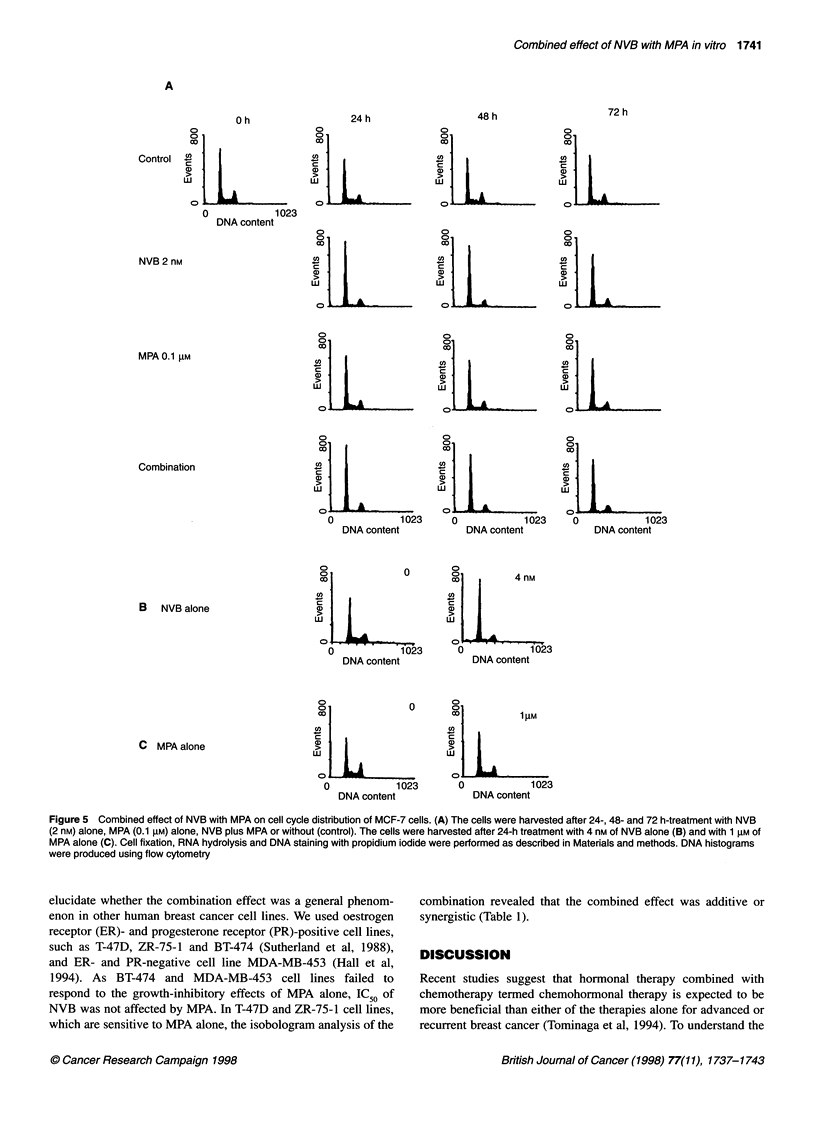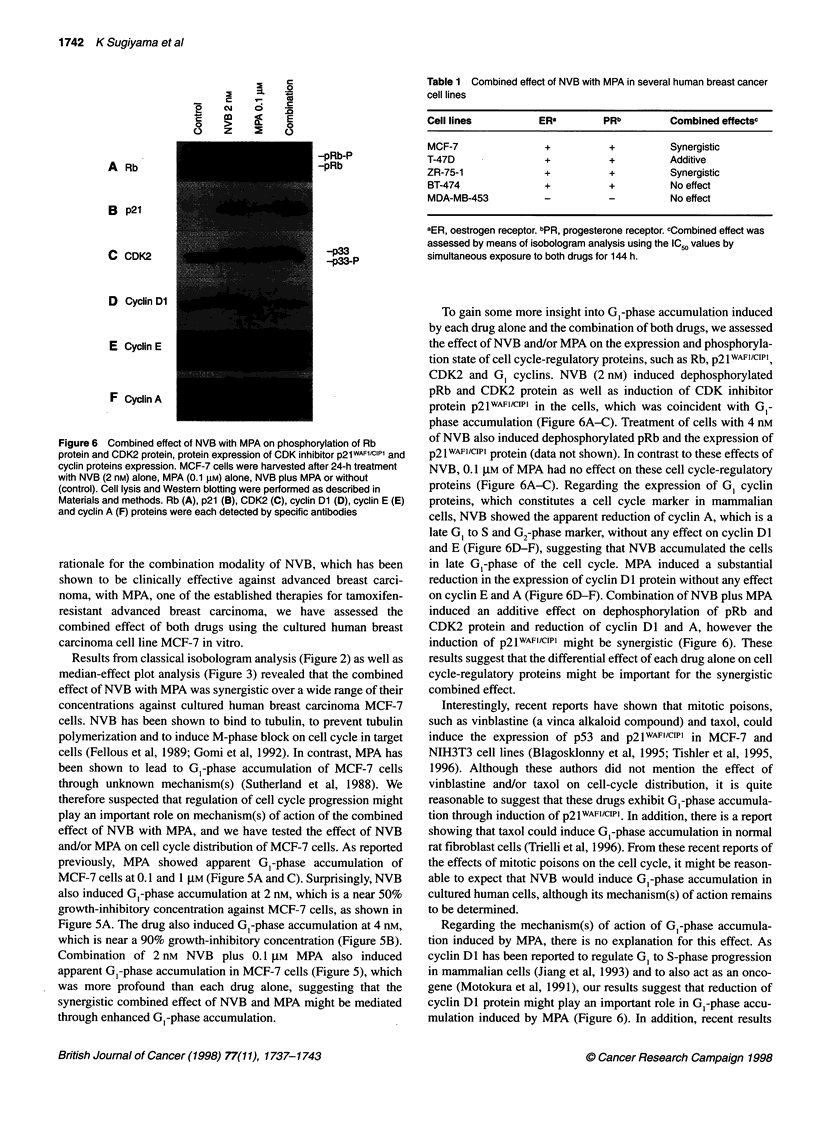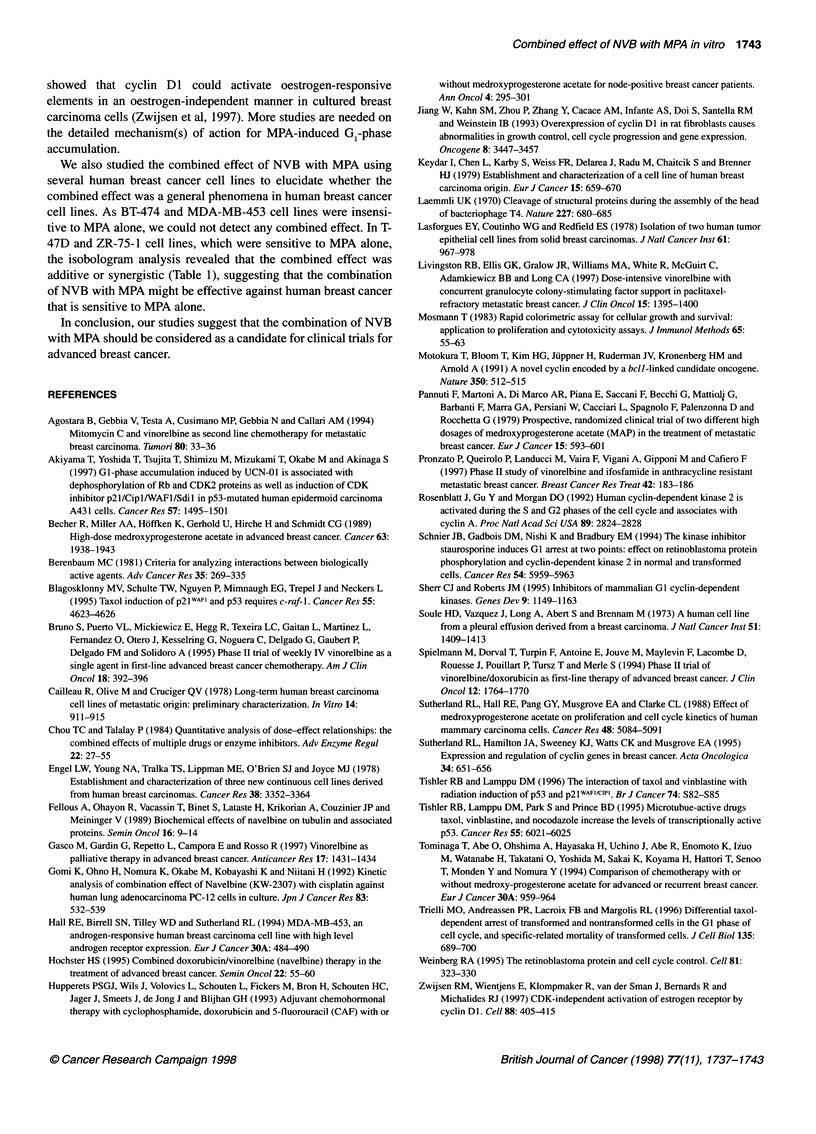# Combined effect of navelbine with medroxyprogesterone acetate against human breast carcinoma MCF-7 cells in vitro.

**DOI:** 10.1038/bjc.1998.291

**Published:** 1998-06

**Authors:** K. Sugiyama, M. Shimizu, T. Akiyama, H. Ishida, M. Okabe, T. Tamaoki, S. Akinaga

**Affiliations:** Pharmaceutical Research Laboratories, Kyowa Hakko Kogyo, Shizuoka, Japan.

## Abstract

**Images:**


British Journal of Cancer (1998) 77(11), 1737–1743
© 1998 Cancer Research Campaign

### Combined effect of navelbine with medroxyprogesterone acetate against human breast carcinoma MCF-7 cells in vitro

**K Sugiyama, M Shimizu, T Akiyama, H Ishida, M Okabe, T Tamaoki and S Akinaga**

Pharmaceutical Research Laboratories, Kyowa Hakko Kogyo, 1188 Shimotogari, Nagaizumi-cho, Sunto-gun, Shizuoka, 411 Japan

**Summary** Navelbine (NVB, vinorelbine ditartrate, KW-2307), a new vinca alkaloid analogue, has been shown to be clinically effective against advanced breast cancer. In this report, the combined effect of NVB with medroxyprogesterone acetate (MPA), a synthetic progesterone derivative, was examined in vitro against human breast carcinoma MCF-7 cells. The combined effect was demonstrated to be synergistic using the isobologram and median-effect plot analyses. To elucidate the mechanism of action, we further examined effects of both drugs on cell cycle distribution of the cells in combination and/or alone. NVB at 2 nM induced apparent $G_1$-phase accumulation as well as the induction of cyclin-dependent kinase (CDK) inhibitor $p21^{WAF1/CIP1}$ protein and the dephosphorylated form of retinoblastoma protein (pRb). In contrast, MPA at 0.1 μM also induced $G_1$-phase accumulation as well as the reduced expression of cyclin D1 protein. In addition, the combination of both drugs induced augmented $G_1$-phase accumulation, which occurred along with $p21^{WAF1/CIP1}$ protein induction, cyclin D1 protein reduction and pRb dephosphorylation. These results demonstrate that the synergistic combined effect of NVB with MPA was mediated through enhancement of $G_1$-phase accumulation that resulted from the different action point(s) of each drug. Furthermore, the synergistic combined effect of NVB with MPA was also observed in other human breast carcinoma cell lines, such as T-47D and ZR-75–1. These results suggest that combination therapy of NVB with MPA in breast cancer might be effective in clinical studies.

**Keywords:** navelbine; medroxyprogesterone acetate; breast cancer; combination effect; $G_1$-phase accumulation

Navelbine (NVB, vinorelbine ditartrate, KW-2307) is a new vinca alkaloid analogue that has been shown to be clinically active against advanced and/or metastatic breast cancer as a single agent (Bruno et al, 1995; Gasco et al, 1997; Livingston et al, 1997). The drug has been also shown to exert objective clinical outcome in combination with other chemotherapeutic agents, such as doxorubicin, mitomycin C and ifosfamide as a first-line or second-line therapy for advanced breast cancer (Agostara et al, 1994; Spielmann et al, 1994; Hochster, 1995; Pronzato et al, 1997).

Medroxyprogesterone acetate (MPA) is a synthetic progesterone derivative that has been shown to be clinically effective against advanced breast cancer as a second-line hormonal therapy after tamoxifen (Pannuti et al, 1979; Becher et al, 1989). MPA has also been shown to augment the efficacy of combined chemotherapeutic modality for breast cancer [such as cyclophosphamide doxorubicin and 5-fluorouracil (CAF)] and to ameliorate side-effects caused by chemotherapeutic agents (Hupperets et al, 1993; Tominaga et al, 1994), thus establishing chemohormonal therapy.

Based on these findings, we have asked whether the combination of NVB with MPA, as a chemohormonal therapy, would be valuable, using cultured human breast carcinoma MCF-7 cells in vitro. Isobologram analysis as well as median-effect plot analysis revealed that the combined effect of NVB with MPA was apparently synergistic. As NVB is known to inhibit the assembly of microtubles, thus inducing mitotic arrest in the cells (Gomi et al, 1992), and MPA is shown to induce $G_1$-phase accumulation (Sutherland et al, 1988), we examined the effect of the combination of both drugs on cell cycle distribution of MCF-7 cells. Unexpectedly, NVB showed $G_1$-phase accumulation both alone and in combination with MPA. Under these circumstances, to examine the mechanism of action of $G_1$-phase accumulation, we have also asked whether NVB and/or MPA affects the expression level of the cell cycle-regulatory proteins such as $G_1$ cyclin and $p21^{WAF1/CIP1}$.

*Received 30 May 1997*
*Accepted 18 August 1997*

*Correspondence to:* S Akinaga

### MATERIALS AND METHODS

### Drugs and reagents

Navelbine (NVB, vinorelbine ditartrate, KW-2307) and medroxyprogesterone acetate (MPA) were obtained from Kyowa Hakko Kogyo, Tokyo, Japan. NVB was dissolved in dimethyl sulphoxide (Wako Pure Chemical Industries, Osaka, Japan). MPA was dissolved in ethanol (Kanto Chemical, Tokyo, Japan). Tris hydroxy aminomethane and Tween 20 were purchased from Bio-Rad Laboratories, Hercules, CA, USA. Sodium chloride, sodium fluoride and ethylenediamine tetra acetic acid (EDTA) were purchased from Kanto Chemical. Triton X-100 was purchased from Yoneyama Yakuhin Kogyo, Osaka, Japan. *N*-2-hydroxyethylpiperazine-*N'*-2-ethanesulphonic acid (HEPES), β-glycerophosphate, sodium o-vanadate, phenylmethylsulphonyl fluoride, aprotinin and leupeptin were purchased from Sigma Chemical, St Louis, MO, USA. Anti-pRb and anti-$p21^{WAF1/CIP1}$ monoclonal antibodies were purchased from PharMingen, San Diego, CA, USA. Anti-cyclin D1 monoclonal antibody was purchased from IBL, Gunma, Japan, and anti-cyclin E monoclonal antibody was purchased from Santa Cruz

Biotechnology, Santa Cruz, CA, USA. Anti-cyclin A and anti-CDK2 monoclonal antibodies were prepared as described previously (Akiyama et al, 1997).

### Cell culture

MCF-7 (Soule et al, 1973), T-47D (Keydar et al, 1979), BT-474 (Lasfargues et al, 1978) and MDA-MB-453 (Cailleau et al, 1978) cells were purchased from the American Type Culture Collection, Rockville, MD, USA. ZR-75-1 cells (Engel et al, 1978) were purchased from the American Type Culture Collection through Dainippon Pharmaceutical, Osaka, Japan. MCF-7, T-47D and ZR-75-1 cells were passaged in RPMI-1640 medium (Gibco, Grand Island, NY, USA) containing 10% fetal bovine serum (Filtron, Brooklyn, Australia), 100 IU penicillin, 100 μg $ml^{-1}$ streptomycin (Gibco) and 10 nM 17β-estradiol (Sigma) at 37°C in a humidified atmosphere containing 5% carbon dioxide in air. BT-474 cells was passaged in the above medium containing 10 μg $ml^{-1}$ insulin (Sigma) at 37°C in a humidified atmosphere containing 5% carbon dioxide in air. MDA-MB-453 cells were passaged in L-15 medium (Sigma) containing 10% fetal bovine serum, 100 IU penicillin and 100 μg $ml^{-1}$ streptomycin at 37°C in a humidified atmosphere.

### Growth-inhibitory activity

MCF-7 cells (5 × $10^3$ per 0.5 ml per well) were precultured in the culture medium for 24 h in 24-well multidishes (Nunc, Roskilde, Denmark). Then the cells were treated with the drug(s) for the period indicated in the text. The drug treatment was terminated by washing the cells with phosphate-buffered saline without calcium [PBS(–)] (ICN Biomedicals, Aurora, OH, USA), and the cells were placed in drug-free medium. The cell number was counted using a micro-cell counter (F-300; Toa Medical Electronics, Hyogo, Japan) after the treatment of the cells with 0.05% trypsin (Difco Laboratories, Detroit, MI, USA)/0.02% EDTA solution.

**Figure 1** Exposure time dependency of the growth-inhibitory activity of NVB (●) and MPA (○). MCF-7 cells (5 × $10^3$ per well) were cultured on day 0 and treated with each drug from day 1 for the indicated exposure time. Cell number was counted on day 7

### Analysis of the combined effect using the isobologram method

Cells were precultured in the culture medium for 24 h in 96-well microwell plates (Nunc). Then, the cells were exposed to drugs according to the following schedules: simultaneous exposure to both drugs for 144 h, sequential exposure to NVB for 24 h followed by MPA for 120 h and sequential exposure to MPA for 120 h followed by NVB for 24 h. Cell viability was determined by MTT assay (Mosmann, 1983). The combination effect of NVB with MPA was assessed by means of isobologram analysis (Berenbaum, 1981).

### Analysis of the combined effect using the median-effect plot method

MCF-7 cells (2.5 × $10^3$ per 0.25 ml per well) were precultured in the culture medium for 24 h in 24-well multidishes. The cells were exposed to both drugs for 144 h. The growth-inhibitory activity was evaluated by counting cell number using a micro-cell counter after separating the cells by treatment with trypsin-EDTA solution. The combined effect of both drugs was determined by median-effect plot analysis (Chou and Talalay, 1984).

### Cell cycle analysis

MCF-7 cells (1.5 × $10^5$ per 10 ml per dish) were precultured in the culture medium for 24 h in a 100-mm culture dish (Falcon 3003; Becton Dickinson, Lincoln Park, NJ, USA). The cells were incubated with the drugs for the indicated time and were harvested by trypsin-EDTA treatment. The cells were then fixed with ice-cold 70% ethanol solution and stored at 4°C. After the cells were washed with PBS(–), they were incubated with PBS(–) containing 250 μg $ml^{-1}$ of ribonuclease A (type 1-A, Sigma), 0.1% Nonidet P-40 (Nacalai Tesque, Kyoto, Japan) for 30 min at 37°C. The cells were stained with propidium iodide (Sigma) at a final concentration of 50 μg $ml^{-1}$ for 20 min on ice. Fluorescence of individual cells was measured with a flow cytometer (Epics Elite, Coulter, Hialeah, FL, USA). The cell cycle distribution was calculated using a Multicycle program (Coulter).

### Western blotting

Exponentially growing MCF-7 cells, exposed to NVB and/or MPA for 24 h, were harvested by treatment with trypsin-EDTA solution, washed with PBS(–) and stored at –80°C. The cells were lysed in lysis buffer [50 mM HEPES/sodium hydroxide (pH 7.4), 150 mM sodium chloride, 0.1% Triton X-100, 50 mM sodium fluoride, 80 mM β-glycerophosphate, 0.1 mM sodium o-vanadate, 1 mM EDTA, 1 mM phenylmethylsulphonyl fluoride, 1 μg $ml^{-1}$ aprotinin, 1 μg $ml^{-1}$ leupeptin] (Rosenblatt et al, 1992) for 20 min at 4°C. The cell lysates were clarified by centrifugation at 14 000 r.p.m. for 10 min at 4°C and the protein contents were determined using the protein assay kit (Bio-Rad Laboratories). Equal amounts of protein were heated in sodium dodecyl sulphate (SDS) sample buffer (Laemmli, 1970) for 5 min at 95°C, subjected to SDS–polyacrylamide gel electrophoresis and transferred onto p-membranes (ATTO, Tokyo, Japan). The membranes were incubated in blocking buffer [5% skim milk (Yukijirushi Nyugyo, Hokkaido, Japan) in Tris-buffered saline], probed with primary antibodies followed by secondary antibody conjugated with horseradish peroxidase

 © Cancer Research Campaign 1998

**Figure 2** The combined effect of NVB with MPA was analysed by the classical isobologram analysis using the $IC_{50}$ (**A–C**) and $IC_{70}$ (**D–F**) values for MCF-7 cells. (**A** and **D**) Simultaneous exposure to both drugs for 144 h. (B and E) Sequential exposure to NVB for 24 h followed by MPA for 120 h. (C and F) Sequential exposure to MPA for 120 h followed by NVB for 24 h

**Figure 3** The combined effect of NVB with MPA was analysed using the median-effect plot analysis for MCF-7 cells. (**A**) The cells (2.5 × $10^3$ per well) were cultured on day 0 and treated with NVB (○), MPA (□) or NVB plus MPA (●) on day 1. For the combination, 1.5-fold dilutions of the two drugs were prepared (0.53–6.08 nM NVB and 10.7–122 nM MPA), and they were combined with each other from the lowest concentration. Drug concentrations were expressed based on NVB concentration. The cell number was counted on day 7. (**B**) Median-effect plot calculated using the results of **A**

(Amersham Life Sciences, Buckinghamshire, UK) and detected using an enhanced chemiluminescence system (Amersham).

### RESULTS

### Kinetic analysis of cell growth-inhibitory activity of NVB and MPA

We carried out kinetic analysis of the cell growth-inhibitory activity of NVB and MPA to determine whether the growth-inhibitory activity of both drugs was AUC dependent or time dependent (Gomi et al, 1992). As shown in Figure 1, NVB showed time-dependent growth-inhibitory characteristics in MCF-7 cells as previously reported. MPA showed plots with a gentle slope, the pattern of which demonstrated neither AUC nor time dependency (Figure 1). From these results we decided to use exposure time as follows to obtain full activity: 24 h for NVB and 144 h for MPA.

### Analysis of the combined effect of NVB with MPA for MCF-7 cells

To determine the combined effect of NVB with MPA, classical isobologram analysis was performed using the $IC_{50}$ (cytostatic condition) and $IC_{70}$ (cytotoxic condition) values for MCF-7 cells. Isobologram analysis on various treatment schedules, such as simultaneous exposure to both drugs (Figure 2A and D), sequential exposure to NVB followed by MPA (Figure 2B and E) and sequential exposure to MPA followed by NVB (Figure 2C and F) revealed that the combination effect of NVB and MPA was synergistic in all the treatment schedules tested. In addition, a synergistic combined effect was observed in both cytostatic and cytotoxic conditions (Figure 2A–C, compared with Figure 2D–F).

The combined effect of NVB with MPA was also assessed using median-effect plot analysis. For the analysis, 1.5-fold dilutions of the two drugs were prepared (0.53–6.08 nM NVB and 10.7–122 nM MPA), and they were combined with each other from the lowest concentration (Figure 3). The combination index (CI) values of the group treated with NVB and MPA were less than 1 at the wide range of concentrations (Figure 3B), indicating that the growth-inhibitory activity in the combination regimen was synergistic (Chou and Talalay, 1984), consistent with the results of the isobologram analysis.

### Growth curve of MCF-7 cells treated with both drugs

To set up the condition for cell cycle analysis, the growth pattern of MCF-7 cells was examined by simultaneous treatment of the cells with NVB and MPA for 144 h. Combination of 0.5 nM NVB with 0.1 μM MPA showed no combined effect (Figure 4A), however the combination of 1 nM or 2 nM NVB with 0.1 μM MPA exhibited synergistic growth inhibition (Figure 4B and C). When 4 nM NVB was combined with 0.1 μM MPA, the growth-inhibitory effect was similar to that of NVB alone (Figure 4D), which is

© Cancer Research Campaign 1998

**Figure 4** Growth curve of MCF-7 cells treated with NVB plus MPA. The cells (5 × 10$^3$ per well) were cultured on day 0, and treated with MPA 0.1 μM, NVB [(**A**) 0.5 nM, (**B**) 1 nM, (**C**) 2 nM and (**D**) 4 nM] or NVB plus MPA from day 1 for 144 h. ○, untreated; □, MPA alone; △, NVB alone; ●, NVB plus MPA

consistent with the results obtained from the median-effect plot analysis.

### Cell cycle analysis

From the results shown in Figure 4, we fixed the concentration of NVB as 2 nM and that of MPA as 0.1 μM for cell cycle analysis. DNA histograms of MCF-7 cells treated with NVB alone, MPA alone or NVB plus MPA are shown in Figure 5. Treatment of MCF-7 cells with 2 nM of NVB alone showed apparent $G_1$-phase accumulation with decrease of cells in S-phase 24 h after the treatment, and the effects persisted until 72 h after drug treatment. The $G_1$-phase accumulation was also observed in the cells treated with 4 nM of NVB alone (Figure 5B). The cells treated with 0.1 μM of MPA alone accumulated in $G_1$-phase 24 h after treatment, and the effects persisted until 72 h; 1 μM of MPA alone also showed $G_1$-phase accumulation in the cells (Figure 5C). In addition, combination of NVB with MPA showed more profound $G_1$-phase accumulation than each drug alone at each time point (Figure 5A). These results suggest that the synergistic combined effect of NVB with MPA might be caused through the augmentation of $G_1$-phase accumulation of each drug alone.

### Combined effect of NVB with MPA on the phosphorylation state of pRb and CDK2 protein and protein expression of CDK inhibitor p21$^{WAF1/CIP1}$

Results from the cell cycle analysis suggested that $G_1$-phase accumulation might play an important role in the combined effect of NVB with MPA. In order to elucidate the mechanism of action of NVB and/or MPA-induced $G_1$-phase accumulation in MCF-7 cells, we have analysed the phosphorylation state of pRb, which plays an important role in $G_1$ to S-phase transition in mammalian cells (Weinberg, 1995). As shown in Figure 6A, in untreated exponentially growing MCF-7 cells, pRb was constitutively hyperphosphorylated and migrated slower than the underphosphorylated form in SDS–polyacrylamide gel (Weinberg, 1995). After a 24 h exposure of the cells to 2 nM of NVB, the accumulation of dephosphorylated, faster migrating pRb was observed (Figure 6A). In contrast, MPA treatment showed no effect on phosphorylation state of pRb. Combination of NVB plus MPA also induced the accumulation of dephosphorylated pRb in the cells, as was the case for NVB alone.

Recent results have shown that vinca alkaloid compounds, such as vinblastine, can induce the expression of CDK inhibitor p21$^{WAF1/CIP1}$ (Tishler et al, 1995). Under these circumstances, we tested whether NVB, which is also a vinca alkaloid compound, could induce the expression of p21$^{WAF1/CIP1}$ in the cells using the Western blotting method. As shown in Figure 6B, NVB did induce the expression of p21$^{WAF1/CIP1}$ protein in the cells (Figure 6B), however MPA showed little, if any, effect on the expression level of p21$^{WAF1/CIP1}$. Combination of NVB with MPA showed more profound induction of p21$^{WAF1/CIP1}$ expression than such each drug alone (Figure 6B).

We also examined the phosphorylation state of CDK2 protein, which is one of the major Rb kinases in mammalian cells (Sherr and Roberts, 1995). As shown in Figure 6C, NVB treatment induced the decrease of the faster migrating protein band that was previously shown to be an active and threonine 160-phosphorylated form of CDK2 protein (Schnier et al, 1994) in MCF-7 cells. However, MPA treatment exhibited no effect on mobility of CDK2 protein in the SDS gel. The combination of NVB with MPA showed the same magnitude of reduction on active CDK2 protein as NVB alone (Figure 6C).

### Combined effect of NVB with MPA on the expression of cyclin proteins

Cyclin D1, E and A proteins have been reported to be expressed and to be degraded depending on cell-cycle phase in breast cancer cells from $G_1$ to S-phase (Sutherland et al, 1995). Namely, cyclin D1 protein is induced at early to mid $G_1$-phase, cyclin E protein is induced at mid to late $G_1$-phase and cyclin A protein induction occurs at late $G_1$-phase to S-phase. To elucidate the arrest point of MCF-7 cells treated with NVB and/or MPA in cell cycle phase, we determined the expression levels of these cyclin proteins after treatment with NVB and/or MPA using the Western blotting method. As reported previously (Sutherland et al, 1995), MCF-7 cells were confirmed to express all the cyclin proteins tested and the expression of cyclin D1 protein was most abundant (Figure 6D–F). Treatment of the cells with 2 nM of NVB for 24 h showed no effect on the expression level of both cyclin D1 and cyclin E protein, while the expression level of cyclin A protein was markedly reduced to a very low or undetectable level (Figure 6D–F). In contrast to these effects induced by NVB, MPA treatment of the cells produced marked reduction of cyclin D1 protein level after 24 h, however the expression levels of cyclin A and cyclin E protein were totally unchanged. Combination of NVB plus MPA produced the reduced expression of cyclin D1 as well as cyclin A protein, to the same extent as MPA or NVB alone without any effect on cyclin E protein level (Figure 6D–F).

### Isobologram analysis of the combined effect of NVB with MPA for several breast cancer cell lines

The combination effect of NVB with MPA was further assessed using isobologram analysis by simultaneous exposure to both drugs for 144 h, using several human breast cancer cell lines, to

 © Cancer Research Campaign 1998

**Figure 5** Combined effect of NVB with MPA on cell cycle distribution of MCF-7 cells. (**A**) The cells were harvested after 24-, 48- and 72 h-treatment with NVB (2 nM) alone, MPA (0.1 μM) alone, NVB plus MPA or without (control). The cells were harvested after 24-h treatment with 4 nM of NVB alone (**B**) and with 1 μM of MPA alone (**C**). Cell fixation, RNA hydrolysis and DNA staining with propidium iodide were performed as described in Materials and methods. DNA histograms were produced using flow cytometry elucidate whether the combination effect was a general phenomenon in other human breast cancer cell lines. We used oestrogen receptor (ER)- and progesterone receptor (PR)-positive cell lines, such as T-47D, ZR-75-1 and BT-474 (Sutherland et al, 1988), and ER- and PR-negative cell line MDA-MB-453 (Hall et al, 1994). As BT-474 and MDA-MB-453 cell lines failed to respond to the growth-inhibitory effects of MPA alone, $IC_{50}$ of NVB was not affected by MPA. In T-47D and ZR-75-1 cell lines, which are sensitive to MPA alone, the isobologram analysis of the combination revealed that the combined effect was additive or synergistic (Table 1).

### DISCUSSION

Recent studies suggest that hormonal therapy combined with chemotherapy termed chemohormonal therapy is expected to be more beneficial than either of the therapies alone for advanced or recurrent breast cancer (Tominaga et al, 1994). To understand the

**Figure 6** Combined effect of NVB with MPA on phosphorylation of Rb protein and CDK2 protein, protein expression of CDK inhibitor p21$^{WAF1/CIP1}$ and cyclin proteins expression. MCF-7 cells were harvested after 24-h treatment with NVB (2 nM) alone, MPA (0.1 μM) alone, NVB plus MPA or without (control). Cell lysis and Western blotting were performed as described in Materials and methods. Rb (**A**), p21 (**B**), CDK2 (**C**), cyclin D1 (**D**), cyclin E (**E**) and cyclin A (**F**) proteins were each detected by specific antibodies

**Table 1** Combined effect of NVB with MPA in several human breast cancer cell lines

| Cell lines | ER[a] | PR[b] | Combined effects[c] |
|---|---|---|---|
| MCF-7 | + | + | Synergistic |
| T-47D | + | + | Additive |
| ZR-75-1 | + | + | Synergistic |
| BT-474 | + | + | No effect |
| MDA-MB-453 | – | – | No effect |

[a]ER, oestrogen receptor. [b]PR, progesterone receptor. [c]Combined effect was assessed by means of isobologram analysis using the $IC_{50}$ values by simultaneous exposure to both drugs for 144 h.

rationale for the combination modality of NVB, which has been shown to be clinically effective against advanced breast carcinoma, with MPA, one of the established therapies for tamoxifen-resistant advanced breast carcinoma, we have assessed the combined effect of both drugs using the cultured human breast carcinoma cell line MCF-7 in vitro.

Results from classical isobologram analysis (Figure 2) as well as median-effect plot analysis (Figure 3) revealed that the combined effect of NVB with MPA was synergistic over a wide range of their concentrations against cultured human breast carcinoma MCF-7 cells. NVB has been shown to bind to tubulin, to prevent tubulin polymerization and to induce M-phase block on cell cycle in target cells (Fellous et al, 1989; Gomi et al, 1992). In contrast, MPA has been shown to lead to $G_1$-phase accumulation of MCF-7 cells through unknown mechanism(s) (Sutherland et al, 1988). We therefore suspected that regulation of cell cycle progression might play an important role on mechanism(s) of action of the combined effect of NVB with MPA, and we have tested the effect of NVB and/or MPA on cell cycle distribution of MCF-7 cells. As reported previously, MPA showed apparent $G_1$-phase accumulation of MCF-7 cells at 0.1 and 1 μM (Figure 5A and C). Surprisingly, NVB also induced $G_1$-phase accumulation at 2 nM, which is a near 50% growth-inhibitory concentration against MCF-7 cells, as shown in Figure 5A. The drug also induced $G_1$-phase accumulation at 4 nM, which is near a 90% growth-inhibitory concentration (Figure 5B). Combination of 2 nM NVB plus 0.1 μM MPA also induced apparent $G_1$-phase accumulation in MCF-7 cells (Figure 5), which was more profound than each drug alone, suggesting that the synergistic combined effect of NVB and MPA might be mediated through enhanced $G_1$-phase accumulation.

To gain some more insight into $G_1$-phase accumulation induced by each drug alone and the combination of both drugs, we assessed the effect of NVB and/or MPA on the expression and phosphorylation state of cell cycle-regulatory proteins, such as Rb, p21$^{WAF1/CIP1}$, CDK2 and $G_1$ cyclins. NVB (2 nM) induced dephosphorylated pRb and CDK2 protein as well as induction of CDK inhibitor protein p21$^{WAF1/CIP1}$ in the cells, which was coincident with $G_1$-phase accumulation (Figure 6A–C). Treatment of cells with 4 nM of NVB also induced dephosphorylated pRb and the expression of p21$^{WAF1/CIP1}$ protein (data not shown). In contrast to these effects of NVB, 0.1 μM of MPA had no effect on these cell cycle-regulatory proteins (Figure 6A–C). Regarding the expression of $G_1$ cyclin proteins, which constitutes a cell cycle marker in mammalian cells, NVB showed the apparent reduction of cyclin A, which is a late $G_1$ to S and $G_2$-phase marker, without any effect on cyclin D1 and E (Figure 6D–F), suggesting that NVB accumulated the cells in late $G_1$-phase of the cell cycle. MPA induced a substantial reduction in the expression of cyclin D1 protein without any effect on cyclin E and A (Figure 6D–F). Combination of NVB plus MPA induced an additive effect on dephosphorylation of pRb and CDK2 protein and reduction of cyclin D1 and A, however the induction of p21$^{WAF1/CIP1}$ might be synergistic (Figure 6). These results suggest that the differential effect of each drug alone on cell cycle-regulatory proteins might be important for the synergistic combined effect.

Interestingly, recent reports have shown that mitotic poisons, such as vinblastine (a vinca alkaloid compound) and taxol, could induce the expression of p53 and p21$^{WAF1/CIP1}$ in MCF-7 and NIH3T3 cell lines (Blagosklonny et al, 1995; Tishler et al, 1995, 1996). Although these authors did not mention the effect of vinblastine and/or taxol on cell-cycle distribution, it is quite reasonable to suggest that these drugs exhibit $G_1$-phase accumulation through induction of p21$^{WAF1/CIP1}$. In addition, there is a report showing that taxol could induce $G_1$-phase accumulation in normal rat fibroblast cells (Trielli et al, 1996). From these recent reports of the effects of mitotic poisons on the cell cycle, it might be reasonable to expect that NVB would induce $G_1$-phase accumulation in cultured human cells, although its mechanism(s) of action remains to be determined.

Regarding the mechanism(s) of action of $G_1$-phase accumulation induced by MPA, there is no explanation for this effect. As cyclin D1 has been reported to regulate $G_1$ to S-phase progression in mammalian cells (Jiang et al, 1993) and to also act as an oncogene (Motokura et al, 1991), our results suggest that reduction of cyclin D1 protein might play an important role in $G_1$-phase accumulation induced by MPA (Figure 6). In addition, recent results

*© Cancer Research Campaign 1998*

showed that cyclin D1 could activate oestrogen-responsive elements in an oestrogen-independent manner in cultured breast carcinoma cells (Zwijsen et al, 1997). More studies are needed on the detailed mechanism(s) of action for MPA-induced $G_1$-phase accumulation.

We also studied the combined effect of NVB with MPA using several human breast cancer cell lines to elucidate whether the combined effect was a general phenomena in human breast cancer cell lines. As BT-474 and MDA-MB-453 cell lines were insensitive to MPA alone, we could not detect any combined effect. In T-47D and ZR-75-1 cell lines, which were sensitive to MPA alone, the isobologram analysis revealed that the combined effect was additive or synergistic (Table 1), suggesting that the combination of NVB with MPA might be effective against human breast cancer that is sensitive to MPA alone.

In conclusion, our studies suggest that the combination of NVB with MPA should be considered as a candidate for clinical trials for advanced breast cancer.

### REFERENCES

Agostara B, Gebbia V, Testa A, Cusimano MP, Gebbia N and Callari AM (1994) Mitomycin C and vinorelbine as second line chemotherapy for metastatic breast carcinoma. *Tumori* **80**: 33–36

Akiyama T, Yoshida T, Tsujita T, Shimizu M, Mizukami T, Okabe M and Akinaga S (1997) G1-phase accumulation induced by UCN-01 is associated with dephosphorylation of Rb and CDK2 proteins as well as induction of CDK inhibitor p21/Cip1/WAF1/Sdi1 in p53-mutated human epidermoid carcinoma A431 cells. *Cancer Res* **57**: 1495–1501

Becher R, Miller AA, Höffken K, Gerhold U, Hirche H and Schmidt CG (1989) High-dose medroxyprogesterone acetate in advanced breast cancer. *Cancer* **63**: 1938–1943

Berenbaum MC (1981) Criteria for analyzing interactions between biologically active agents. *Adv Cancer Res* **35**: 269–335

Blagosklonny MV, Schulte TW, Nguyen P, Mimnaugh EG, Trepel J and Neckers L (1995) Taxol induction of p21$^{WAF1}$ and p53 requires *c-raf-*1. *Cancer Res* **55**: 4623–4626

Bruno S, Puerto VL, Mickiewicz E, Hegg R, Texeira LC, Gaitan L, Martinez L, Fernandez O, Otero J, Kesselring G, Noguera C, Delgado G, Gaubert P, Delgado FM and Solidoro A (1995) Phase II trial of weekly IV vinorelbine as a single agent in first-line advanced breast cancer chemotherapy. *Am J Clin Oncol* **18**: 392–396

Cailleau R, Olive M and Cruciger QV (1978) Long-term human breast carcinoma cell lines of metastatic origin: preliminary characterization. *In Vitro* **14**: 911–915

Chou TC and Talalay P (1984) Quantitative analysis of dose–effect relationships: the combined effects of multiple drugs or enzyme inhibitors. *Adv Enzyme Regul* **22**: 27–55

Engel LW, Young NA, Tralka TS, Lippman ME, O'Brien SJ and Joyce MJ (1978) Establishment and characterization of three new continuous cell lines derived from human breast carcinomas. *Cancer Res* **38**: 3352–3364

Fellous A, Ohayon R, Vacassin T, Binet S, Lataste H, Krikorian A, Couzinier JP and Meininger V (1989) Biochemical effects of navelbine on tubulin and associated proteins. *Semin Oncol* **16**: 9–14

Gasco M, Gardin G, Repetto L, Campora E and Rosso R (1997) Vinorelbine as palliative therapy in advanced breast cancer. *Anticancer Res* **17**: 1431–1434

Gomi K, Ohno H, Nomura K, Okabe M, Kobayashi K and Niitani H (1992) Kinetic analysis of combination effect of Navelbine (KW-2307) with cisplatin against human lung adenocarcinoma PC-12 cells in culture. *Jpn J Cancer Res* **83**: 532–539

Hall RE, Birrell SN, Tilley WD and Sutherland RL (1994) MDA-MB-453, an androgen-responsive human breast carcinoma cell line with high level androgen receptor expression. *Eur J Cancer* **30A**: 484–490

Hochster HS (1995) Combined doxorubicin/vinorelbine (navelbine) therapy in the treatment of advanced breast cancer. *Semin Oncol* **22**: 55–60

Hupperets PSGJ, Wils J, Volovics L, Schouten L, Fickers M, Bron H, Schouten HC, Jager J, Smeets J, de Jong J and Blijhan GH (1993) Adjuvant chemohormonal therapy with cyclophosphamide, doxorubicin and 5-fluorouracil (CAF) with or without medroxyprogesterone acetate for node-positive breast cancer patients. *Ann Oncol* **4**: 295–301

Jiang W, Kahn SM, Zhou P, Zhang Y, Cacace AM, Infante AS, Doi S, Santella RM and Weinstein IB (1993) Overexpression of cyclin D1 in rat fibroblasts causes abnormalities in growth control, cell cycle progression and gene expression. *Oncogene* **8**: 3447–3457

Keydar I, Chen L, Karby S, Weiss FR, Delarea J, Radu M, Chaitcik S and Brenner HJ (1979) Establishment and characterization of a cell line of human breast carcinoma origin. *Eur J Cancer* **15**: 659–670

Laemmli UK (1970) Cleavage of structural proteins during the assembly of the head of bacteriophage T4. *Nature* **227**: 680–685

Lasforgues EY, Coutinho WG and Redfield ES (1978) Isolation of two human tumor epithelial cell lines from solid breast carcinomas. *J Natl Cancer Inst* **61**: 967–978

Livingston RB, Ellis GK, Gralow JR, Williams MA, White R, McGuirt C, Adamkiewicz BB and Long CA (1997) Dose-intensive vinorelbine with concurrent granulocyte colony-stimulating factor support in paclitaxel-refractory metastatic breast cancer. *J Clin Oncol* **15**: 1395–1400

Mosmann T (1983) Rapid colorimetric assay for cellular growth and survival: application to proliferation and cytotoxicity assays. *J Immunol Methods* **65**: 55–63

Motokura T, Bloom T, Kim HG, Jüppner H, Ruderman JV, Kronenberg HM and Arnold A (1991) A novel cyclin encoded by a *bcl1*-linked candidate oncogene. *Nature* **350**: 512–515

Pannuti F, Martoni A, Di Marco AR, Piana E, Saccani F, Becchi G, Mattioli G, Barbanti F, Marra GA, Persiani W, Cacciari L, Spagnolo F, Palenzonna D and Rocchetta G (1979) Prospective, randomized clinical trial of two different high dosages of medroxyprogesterone acetate (MAP) in the treatment of metastatic breast cancer. *Eur J Cancer* **15**: 593–601

Pronzato P, Queirolo P, Landucci M, Vaira F, Vigani A, Gipponi M and Cafiero F (1997) Phase II study of vinorelbine and ifosfamide in anthracycline resistant metastatic breast cancer. *Breast Cancer Res Treat* **42**: 183–186

Rosenblatt J, Gu Y and Morgan DO (1992) Human cyclin-dependent kinase 2 is activated during the S and G2 phases of the cell cycle and associates with cyclin A. *Proc Natl Acad Sci USA* **89**: 2824–2828

Schnier JB, Gadbois DM, Nishi K and Bradbury EM (1994) The kinase inhibitor staurosporine induces G1 arrest at two points: effect on retinoblastoma protein phosphorylation and cyclin-dependent kinase 2 in normal and transformed cells. *Cancer Res* **54**: 5959–5963

Sherr CJ and Roberts JM (1995) Inhibitors of mammalian G1 cyclin-dependent kinases. *Genes Dev* **9**: 1149–1163

Soule HD, Vazquez J, Long A, Abert S and Brennam M (1973) A human cell line from a pleural effusion derived from a breast carcinoma. *J Natl Cancer Inst* **51**: 1409–1413

Spielmann M, Dorval T, Turpin F, Antoine E, Jouve M, Maylevin F, Lacombe D, Rouesse J, Pouillart P, Tursz T and Merle S (1994) Phase II trial of vinorelbine/doxorubicin as first-line therapy of advanced breast cancer. *J Clin Oncol* **12**: 1764–1770

Sutherland RL, Hall RE, Pang GY, Musgrove EA and Clarke CL (1988) Effect of medroxyprogesterone acetate on proliferation and cell cycle kinetics of human mammary carcinoma cells. *Cancer Res* **48**: 5084–5091

Sutherland RL, Hamilton JA, Sweeney KJ, Watts CK and Musgrove EA (1995) Expression and regulation of cyclin genes in breast cancer. *Acta Oncologica* **34**: 651–656

Tishler RB and Lamppu DM (1996) The interaction of taxol and vinblastine with radiation induction of p53 and p21$^{WAF1/CIP1}$. *Br J Cancer* **74**: S82–S85

Tishler RB, Lamppu DM, Park S and Prince BD (1995) Microtubue-active drugs taxol, vinblastine, and nocodazole increase the levels of transcriptionally active p53. *Cancer Res* **55**: 6021–6025

Tominaga T, Abe O, Ohshima A, Hayasaka H, Uchino J, Abe R, Enomoto K, Izuo M, Watanabe H, Takatani O, Yoshida M, Sakai K, Koyama H, Hattori T, Senoo T, Monden Y and Nomura Y (1994) Comparison of chemotherapy with or without medroxy-progesterone acetate for advanced or recurrent breast cancer. *Eur J Cancer* **30A**: 959–964

Trielli MO, Andreassen PR, Lacroix FB and Margolis RL (1996) Differential taxol-dependent arrest of transformed and nontransformed cells in the G1 phase of cell cycle, and specific-related mortality of transformed cells. *J Cell Biol* **135**: 689–700

Weinberg RA (1995) The retinoblastoma protein and cell cycle control. *Cell* **81**: 323–330

Zwijsen RM, Wientjens E, Klompmaker R, van der Sman J, Bernards R and Michalides RJ (1997) CDK-independent activation of estrogen receptor by cyclin D1. *Cell* **88**: 405–415

© Cancer Research Campaign 1998